# Xeno-Free Defined Conditions for Culture of Human Embryonic Stem Cells, Neural Stem Cells and Dopaminergic Neurons Derived from Them

**DOI:** 10.1371/journal.pone.0006233

**Published:** 2009-07-14

**Authors:** Andrzej Swistowski, Jun Peng, Yi Han, Anna Maria Swistowska, Mahendra S. Rao, Xianmin Zeng

**Affiliations:** Buck Institute for Age Research, Novato, California, United States of America; University of North Dakota, United States of America

## Abstract

**Background:**

Human embryonic stem cells (hESCs) may provide an invaluable resource for regenerative medicine. To move hESCs towards the clinic it is important that cells with therapeutic potential be reproducibly generated under completely defined conditions.

**Methodology/Principal Findings:**

Here we report a four-step scalable process that is readily transferable to a Good Manufacture Practice (GMP) facility for the production of functional dopaminergic neurons from hESCs for potential clinical uses. We show that each of the steps (propagation of ESC→generation of neural stem cells (NSC)→induction of dopaminergic precursors→maturation of dopaminergic neurons) could utilize xeno-free defined media and substrate, and that cells could be stored at intermediate stages in the process without losing their functional ability. Neurons generated by this process expressed midbrain and A9 dopaminergic markers and could be transplanted at an appropriate time point in development to survive after transplant.

**Conclusions/Significance:**

hESCs and NSCs can be maintained in xeno-free defined media for a prolonged period of time while retaining their ability to differentiate into authentic dopaminergic neurons. Our defined medium system provides a path to a scalable GMP-applicable process of generation of dopaminergic neurons from hESCs for therapeutic applications, and a ready source of large numbers of neurons for potential screening applications.

## Introduction

Pluripotent human embryonic stem cells (hESCs) have the capacity to differentiate into all of the somatic cell types and therefore hold great promise for regenerative medicine. One key issue that needs to be addressed in guiding hESC technology from “bench” toward “bedside” is developing defined cell culture systems for culture of hESCs, and differentiation of such cells into therapeutically relevant cells using clinically compliant systems. Early hESC culture systems typically include the use of mouse embryonic fibroblast (MEF) feeders or medium conditioned on MEFs in the presence of serum or serum substitutes such as knockout serum replacement [1]–[3]. Human feeders and serum have also been used for hESC culture [4], however, the use of serum or serum replacement which contains undefined xenogenic factors in such cultures is still an issue for potential clinical applications. Recent advances in the identification of multiple factors that play a role in sustaining pluripotency have led to the report of several defined medium systems for hESC culture and derivation [5]–[9]. These defined media are based upon the use of FGF2 in combination with TGFβ/nodal/activin signaling molecules, IGF1R or N2/B27 supplements, and do not include serum or serum replacement. Two such defined media (TeSR1 from Stem Cell Technology and StemPro from Invitrogen) are currently commercially available.

Despite this progress in the development of defined medium systems for culturing hESCs, little is known about whether hESCs derived in defined media or cultured in defined media for an extended period can differentiate into functional cells of therapeutic relevance under xeno-free defined conditions. Indeed, few defined culture systems have been described for lineage-specific differentiation, despite the fact that cells with potential therapeutic uses including neurons, cardiomyocytes and insulin-producing beta cells have been successfully generated from hESCs. Most of the current protocols include the step of embryoid body (EB) formation with serum or serum replacement, which introduces components that are not chemically defined. Thus, further efforts are warranted to define robust culture systems for lineage-specific differentiation of hESCs adapted to or derived from defined medium into various therapeutically relevant somatic cells.

Of particular interest are midbrain dopaminergic neurons, the population of neurons selectively lost in Parkinson's disease (PD). Since the motor symptoms of PD are a consequence of focal damage to dopaminergic neurons of substantia nigra, it has been widely suggested that implantation of a relatively small number of dopaminergic neurons may restore functionality in PD patients. In the past three decades, it has been demonstrated that grafts of dopaminergic neurons derived from fetal and/or embryonic cells survive, reinnervate appropriate targets, and function in vivo in rodent and non-human primate PD models [10]–[15]. Proof of principle for cell replacement therapy in PD has also been achieved in several clinical trials by using fetal derived dopaminergic neurons [16]–[19]. Contemporary work has shown that authentic dopaminergic neurons can be generated from hESCs, which may provide an unlimited source of cells for transplantation, and grafts of these cells can release dopamine and ameliorate behavioral deficits in rodent PD models [20]–[22]. We believe the time is right for the development of scalable protocols for generating dopaminergic neurons from hESCs using completely defined, xeno-free components. This approach will facilitate subsequent adaptation of protocols to Good Manufacturing Practice (GMP) standards which is a pre-requisite for progression towards clinical trials.

In pursuing this goal, we have developed a GMP-compliant scalable process of generating neural stem cells (NSCs) and further differentiate them into dopaminergic neurons in defined conditions from hESCs adapted to a defined medium. We show that functional dopaminergic neurons can be generated under completely defined conditions from hESCs adapted to a defined medium system. We believe our system not only provides a platform for producing therapeutic cells from hESCs for PD patients, but also a generalized plan for how hESC culture and lineage-specific differentiation need to be developed for treatment of other degenerative disorders, including spinal cord injury and type I diabetes.

## Materials and Methods

### Cell culture

hESC line I6 at passage 42 was adopted to serum-free StemPro hESC SFM defined medium and maintained on culture dishes coated with xeno-free CellStart or Geltrex (All from Invitrogen; Carlsbad, CA). The medium comprises of DMEM/F12 with GlutaMax, BSA 1.8%, β-Mercaptoethanol 0.1 mM, 1× StemPro protein cocktail (All from Invitrogen) and 8 ng/ml of basic fibroblast growth factor (FGF2, Sigma). Cells were split every 4–6 days using a cell scraper.

### Generation and prolonged culture of NSCs

To derive NSCs, hESC colonies were harvested using a scraper with care taken to avoid high colony fragmentation. Colonies were cultured in suspension as EBs on Petri dishes with agitation for 8 days in StemPro defined media minus FGF2. EBs were then cultured for additional 2–3 days in suspension in neural induction media containing DMEM/F12 with Glutamax, 1×NEAA, 1×N2 and FGF2 (20 ng/ml) prior to attachment on cell culture plates coated with CellStart. Numerous neural rosettes were formed 2–3 days after adherent culture. To obtain a pure population of NSCs, rosettes were manually isolated using stretched glass Pasteur pipette and placed in fresh culture dishes. The rosettes were then dissociated into single cells using accutase and replated onto culture dishes to obtain a homogeneous population of NSCs. The NSCs population was expanded in Neurobasal media containing 1×NEAA, 1×L-Glutamine (2 mM), 1×B27, LIF, FGF2 20 ng/ml.

To confirm that NSCs can differentiate into astrocytes, NSCs were cultured in DMEM/F12 medium supplemented with 1×NEAA, L-Glutamine (2 mM), 1×N2 and 1×B27 for two weeks and processed for immunostaining. To initiate oligodendrocyte differentiation, NSCs were cultured in medium containing DMEM/F12, 1×NEAA, 1×L-Glutamine (2 mM), 1×N2, 1×B27, Shh (200 ng/ml, R&D Systems) and retinoic acid (RA, 2 µM) for 10 days, and then with T3 (30 ng/ml, R&D Systems) and PDGFα (10 ng/ml, R&D Systems) but without Shh and RA for an additional 2 weeks.

### Dopaminergic differentiation of NSCs

Dopaminergic differentiation was obtained by culturing NSCs in medium conditioned on PA6 cells (PA6-CM) [23] for 3–6 weeks, or in xeno-free defined media for 4–5 weeks on culture dishes or glass cover slips coated with Poly-L-ornithine (20 ug/ml) and laminin (10 ug/ml). Dopaminergic differentiation in defined media was initiated by culturing NSCs for 10 days in Neurobasal medium supplemented with 1×NEAA, 1×L-Glutamine (2 mM), 1×B27 (Gibco), Shh (200 ng/ml, R&D Systems) and FGF8 (50–100 ng/ml, R&D Systems). Shh and FGF8 were then withdrawn and replaced with BDNF (20 ng/ml, R&D Systems) and GDNF (20 ng/ml, R&D Systems), TGF-β3 (1 µM, R&D Systems) and dcAMP (1 mM, Sigma) for 10–25 days.

### Immunocytochemistry

Immunocytochemistry and staining procedures were as described previously [24]. Briefly, hESCs at different stages of dopaminergic differentiation were fixed with 2% paraformaldehyde for half an hour. Fixed cells were blocked in blocking buffer (10% goat serum, 1% BSA, 0.1% Triton X-100) for 1 hour followed by incubation with the primary antibody at 4°C overnight in 8% goat serum, 1% BSA, 0.1% Triton X-100. Appropriately coupled secondary antibodies (Molecular Probes) were used for single and double labeling. All secondary antibodies were tested for cross reactivity and non-specific immunoreactivity. The following primary antibodies were used: Oct4 (ab19857 AbCam) 1∶1000; β-Tubulin isotype III clone SDL.3D10 (T8660 Sigma) 1∶500; GalC (MAB342 Millipore) 1∶50; GFAP (M0761 Chemicon) 1∶50; Nestin (611658 BD Transduction laboratories) 1∶500; Sox1 (AB5768 Chemicon) 1∶200, TH (Pel-Freeze P40101) 1∶500, TH clone TH-16 (T2928 Sigma) 1∶1000; Lmx1a (a kind gift from Dr. Michael German, UCSF) 1∶1500, Girk2 (AB5200 Chemicon) 1∶250; Vmat2 (PPS089 R&D) 1∶1000, GABA (ab8891 Abcam) 1∶500, L-Glutamate (ab8889 Abcam) 1∶500, DBH (AB1585 Chemicon) 1∶1000, HB9 (81.5C10 DSHB) 1∶50, and as secondary antibodies: Alexa Fluor 488 Goat Anti-Mouse, Alexa Fluor 594 Goat Anti-Mouse, Alexa Fluor 488 Goat Anti-Rabbit, Alexa Fluor 594 Goat Anti-Rabbit. Hoechst 33342 (Molecular Probes H3570) 1∶1000 was used for nuclei identification. Images were captured on a Nikon fluorescence microscope.

The quantification of TH^+^ cells in culture was performed by analyzing fluorescent images using Photoshop on a minimum of 5000 cells of at least 10 randomly chosen fields derived from 3 or more independent experiments. The number of Hoechst labeled nuclei on each image was referred as total cell number (100%).

### Gene expression and PCR analysis

RNAs isolated from NSCs and dopaminergic populations were hybridized to Illumina HumanRef-8 BeadChip (Illumina, Inc, performed by Microarray core facility at the Burnham Institute for Medical Research). The Illumina array data were normalized by the quantile method, and then transformed log2 ratio values for a zero mean for expression values of each gene across all samples. The bioinformatics and statistical analyses were conducted by using R and the bioconductor package (www.bioconductor.org).

cDNA was synthesized by using a reverse transcription kit SuperScript III First-Strand Synthesis System for RT-PCR (Invitrogen) according to the manufacturer's recommendations. Real-time PCR was used to quantify the levels of mRNA expression of 10 genes in NSC and day 16 and 31 dopaminergic populations. PCR reactions were carried out by ABI 900HT instrument according to the manufacturer's instructions. All qRT-PCR primer sequences are shown in supplementary Table S1.

### Transplantation of hESC-derived dopaminergic neurons into rat brains and histological analysis

Adult female Sprague-Dawley rats (200–230 g) were purchased from Charles River Laboratories (Wilmington, MA, USA). Four rats were injected with day 20 (after the NSC stage) hESC-derived neuronal populations (approximately 500,000 cells in 5 µl of cell preparation medium) or vehicle with a Hamilton syringe into the striatum (−0.3 mm anterior to bregma, 3.0 mm lateral to midline, and 5.0 mm beneath dura). Cyclosporine A (10 mg/kg; Novartis Pharmaceuticals) was intraperitoneally injected 24 hours before transplantation and every day afterwards until the rats were sacrificed. Experimental protocols were in accordance with the National Institutes of Health Guidelines for Use of Live Animals and were approved by the Animal Care and Use Committee at the Buck Institute. Rats were perfused with phosphate-buffered saline (PBS) followed by 4% paraformaldehyde.

For histological analysis, brains were removed and immersion-fixed overnight at room temperature. Brains were then dehydrated in graded ethanols, cleared in xylene, and paraffin-embedded [25]. Seven µm-thick serial coronal sections were cut and mounted on glass slides, which were dried overnight at 42°C. Sections were deparaffinized, rehydrated through a graded series of ethanols, and washed in water. For immunostaining [26], sections were incubated with blocking solution (2% horse serum, 1% bovine serum albumin, and 0.1% Triton X-100 in phosphate-buffered saline, pH 7.5) and then with primary antibodies at 4°C overnight followed by secondary antibodies in blocking solution at room temperature for 2 h. The primary antibodies used were rabbit anti-TH polyclonal antibody (Chemicon, 1∶500) and mouse polyclonal anti-human nuclear antigen (Chemicon, 1∶300). The secondary antibodies were rhodamine-conjugated rat-absorbed donkey anti-rabbit IgG (Jackson ImmunoResearch; 1∶200) and fluorescein isothiocyanate-conjugated pig anti-mouse IgG (Vector Laboratories; 1∶200). Nuclei were counterstained with 4′,6-diamidino-2-phenylindole (DAPI) using proLong Gold anti-fade reagent (Invitrogen). To quantitatively analyze double labeled neurons in the striatum, fluorescence signals were detected with an LSM 510 NLO Confocal Scanning System mounted on an Axiovert 200 inverted microscope (Carl Zeiss Ltd.) equipped with a two-photon Chameleon laser (Coherent Inc.). Three-color images were scanned using Argon and 543 HeNe lasers. IMARIS (Bitplane AG.) imaging software was used for three-dimensional image reconstruction. Images were acquired using LSM 510 Imaging Software (Carl Zeiss Ltd.) as described previously [27]. The specificity of each label was first verified using single-channel scans that were then merged into multiple-channel views. Neurons were considered double-labeled if co-labeling with relevant morphology was seen throughout the extent of the nucleus for nuclear markers or if a cytoplasmic marker surrounds a nuclear marker when viewed in *x-y* cross section as well as in *x-z* and *y-z* cross-sections produced by orthogonal reconstructions from *z*-stacks taken at 400× magnification. Human antigen single-labeled neurons and neurons double labeled for TH and human antigen were recorded in every seventh section per animal (n = 3). The area of each transplanted region was simultaneously determined for each of the scored sections.

### Transplantation into the 6-hydroxydopamine rats and behavior analysis

Thirty rats were anesthetized and 16 µg of 6-hydroxydopamine (6-OHDA) (Sigma-Aldrich) were stereotaxically injected at a concentration of 4 µg/µl (in 0.2 mg/ml ascorbate in saline) at one site in the MFB [stereotaxic coordinates: anteroposterior (AP), −4.4 mm; mediolateral (ML), −1.2 mm; dorsoventral (DV), −7.8 mm] using a Hamilton syringe. The toxin was injected at a rate of 1 µl/min. To ensure complete lesion of the nigrostriatal DA pathway, the animals were screened by amphetamine-induced rotation at a dose of 2.5 mg/kg. Only animals (26) that exhibited a mean ipsilateral rotation score of seven or more complete body turns per minute were included in the study.

For rational behavior analysis the animals were given 2.5 mg/kg D-amphetamine intraperitoneally and their rotational behavior was monitored over a 90 min period using the TSE rotameter system (Bad Homburg, Germany). Rotation toward the lesion (ipsilateral) was scored as positive and net rotational asymmetry score were expressed as full body turns per minute.

## Results

### NSCs can be generated from hESCs adapted to a serum-free defined condition and a defined substrate

**Figure 1:**
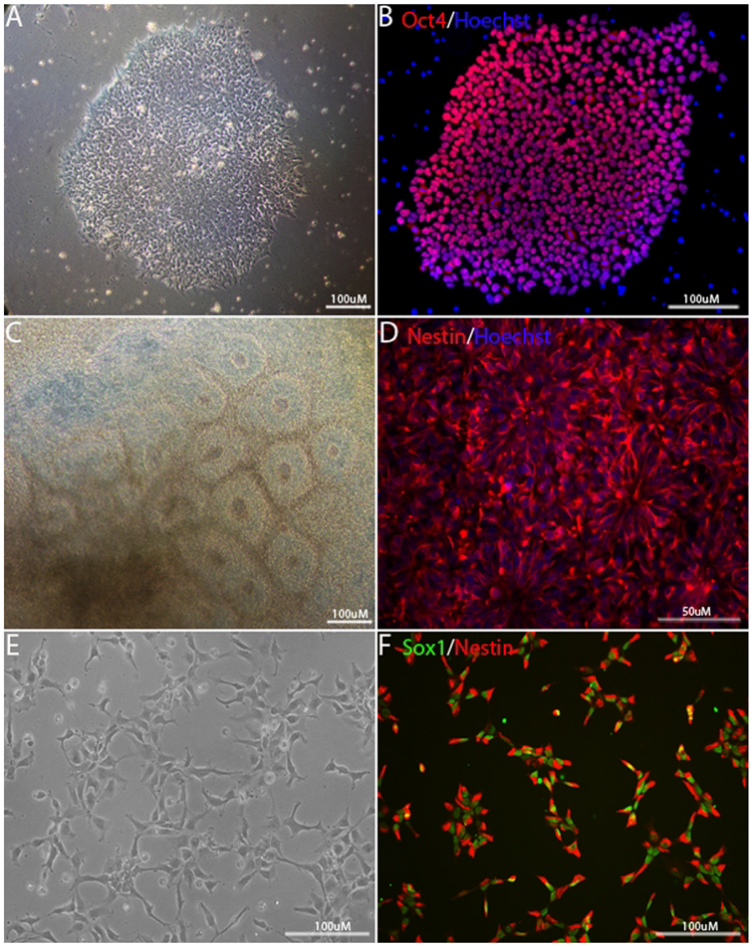
Generation of NSCs from hESCs adapted to defined medium. hESC line I6 at passage 42 was adapted to a chemically defined medium StemPro. (A–B) Morphology (A) and expression of the pluripotent marker Oct4 (B) in hESCs that were cultured in StemPro for 28 passages. (C–F) Generation of NSCs in defined conditions. Nestin^+^ neural tube-like rosette structures were formed in the center of the ESC colonies after 12–14 days of differentiation (C–D). A monolayer of homogeneous NSCs expressed Sox1 and nestin (E–F).

We have previously reported the culture of hESCs in a serum-free, completely chemically defined medium [28] and wished to test whether cells adapted to such defined conditions can give rise to NSCs. We employed two hESC lines (I6 and W10) and, since the results were similar, only the results from one of the lines (I6) are reported here. I6 cells were continuously cultured in this defined medium (StemPro ESC defined medium) for 6 months on a defined substrate (CellStart). As seen in Fig. 1A–B, hESCs cultured in defined medium and substrate showed typical undifferentiated morphology without differentiated cells surrounding the colonies, and expressed the pluripotency marker Oct4. No karyotypical abnormality was detected in cultures after over 25 passages (data not shown).

To generate NSCs, ESC colonies grown in defined medium were detached and cultured in suspension as EBs in ESC defined medium without FGF2 for 8 days. EBs were directed towards neural lineages by the addition of FGF2 and allowed to attach in adherent cultures in a defined serum-free medium. After 2–3 days of attachment, numerous neural tube-like rosette structures were formed in the center of the ESC colonies (Fig. 1C), which were immuno-positive for nestin (Fig. 1D). The rosettes were manually dissected and expanded in NSC proliferation medium on defined substrate (Fig. 1E). Morphologically, these cells were homogenous and uniformly expressing nestin and Sox1, a transcription factor expressing in early neuroepithelial cells (Fig. 1F). Few cells (less than 1%) expressed β-III tubulin and no cells were positive for GFAP or O4 (data not shown). We noted that several radial glia markers such as Glast, Vim and Blbp were upregulated in our NSC population (Expression level measured by intensity for Glast, Vim and Blbp are 433, 16719 and 1273, respectively) compared to undifferentiated ESCs (Expression level measured by intensity for Glast, Vim and Blbp are 209, 10065 and 200, respectively) by global gene expression using Illumina array, consistent with the observation by Nat et al. that radial glia markers were expressed in ESC-derived NSCs [29]. These results indicate that NSCs can be generated in defined conditions from hESCs adapted to defined conditions.

### Prolonged culture of NSCs in defined conditions does not diminish their ability to differentiate

**Figure 2:**
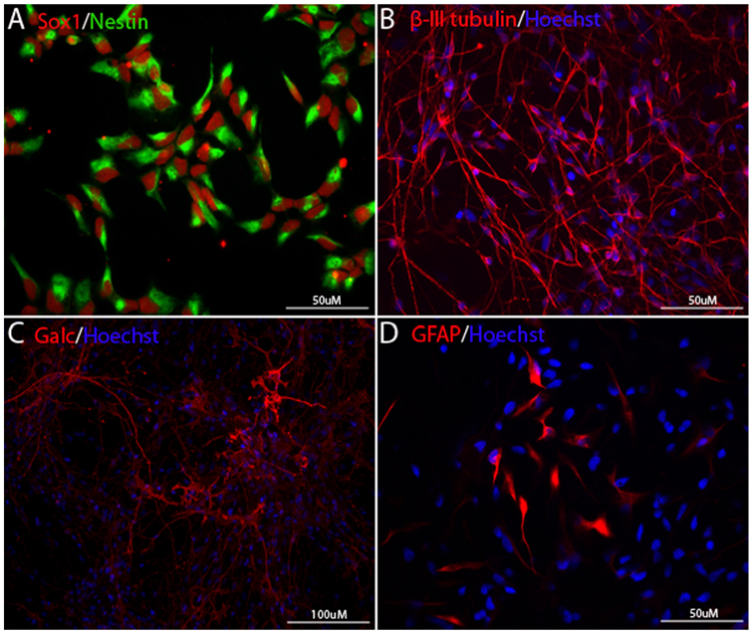
NSCs cultured in xeno-free defined medium for a prolonged period remain to be multipotent. (A) NSCs propagated in defined conditions for 15 passages retained NSC identity as evident by expressing Sox1 and nestin. (B–C) NSCs can be frozen and thawed and maintained the capacity to differentiate into neurons (B), oligodendrocytes (C) and astrocytes (D).

To test whether NSCs could be propagated in a defined medium for a prolonged period and retain the capacity to differentiate into neurons and glia, we continuously passaged the cells in the defined medium (NSC medium) on defined substrate for over 2 months. As seen in Fig. 2A, NSCs propagated in defined conditions for 15 passages expressed Sox1 and nestin, and differentiated into β-III-tubulin^+^ neurons, GFAP^+^ astrocytes and GalC^+^ oligodendrocytes in conditions promoting differentiation (Fig. 2B–C). In addition, these cells could be frozen and thawed with greater than 95% viability and without losing the ability to differentiate into neurons (including dopaminergic neurons) and glia.

### NSCs cultured for prolonged periods retain their ability to differentiate into dopaminergic neurons upon exposure to PA6-CM or in a xeno-free defined medium

**Figure 3:**
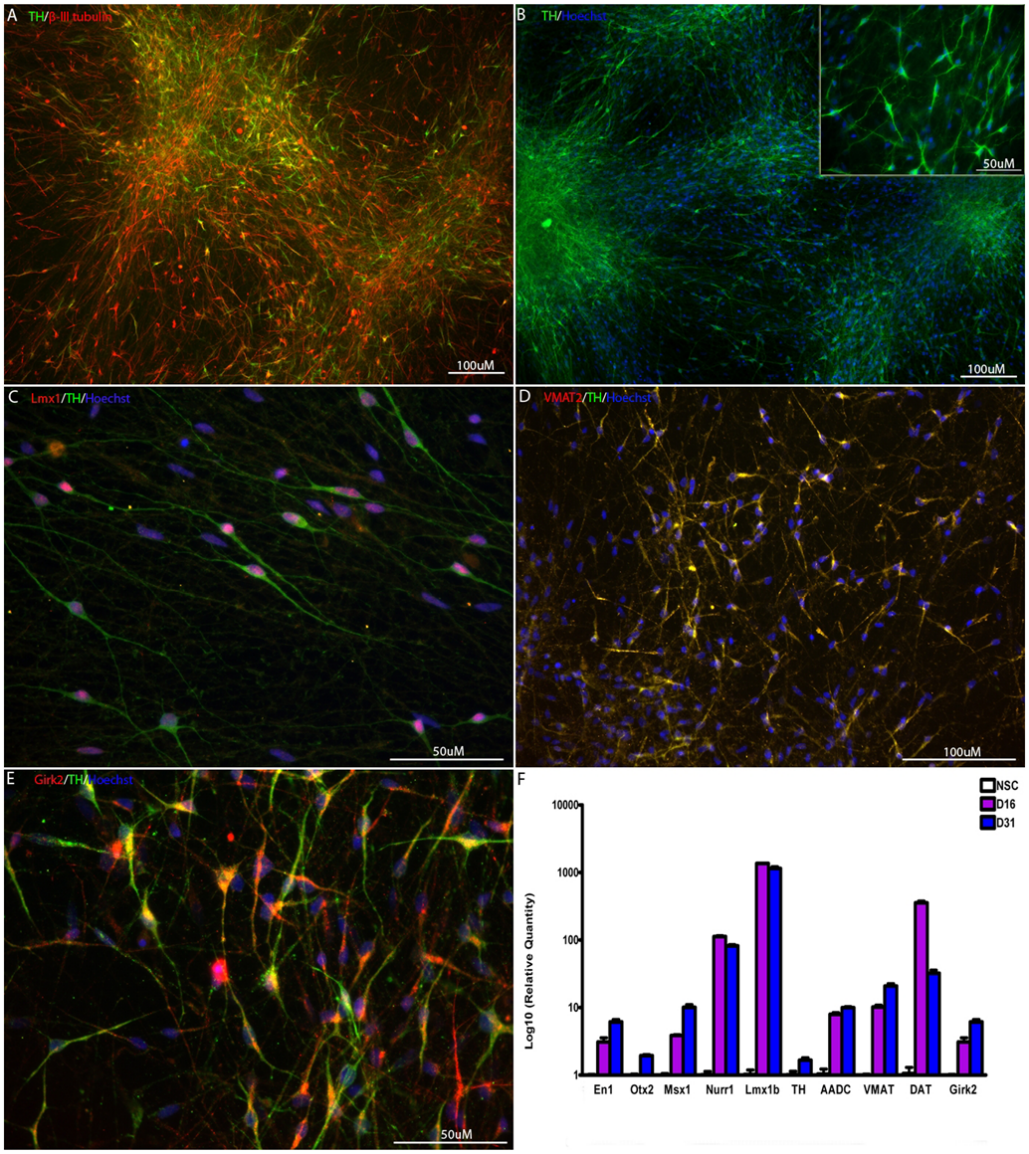
NSCs from prolonged culture in defined conditions can be efficiently differentiated into midbrain dopaminergic neurons. (A–E) Efficient differentiation into midbrain dopaminergic neurons by PA6-CM as shown by immunocytochemistry. The majority of the cells expressed β-III-tubulin and TH after 4 weeks of differentiation in PA6-CM (A–B). Co-expression of midbrain and A9 markers in TH^+^ dopaminergic neurons: Lmx1a (C), VMAT (D) and Girk2 (E). (F) Differential expression of dopaminergic markers in several stages of differentiation (NSC, dopaminergic precursors and dopaminergic neurons) by quantitative PCR. All the examined markers were up-regulated in dopaminergic populations compared to NSCs.

Our laboratory has reported dopaminergic inducing activity of medium conditioned on stromal cell line PA6 (PA6-CM) and shown that exposure to PA6-CM at a defined time period (NSC stage) was sufficient for a dopaminergic fate [23]. We therefore tested whether NSCs grown to passages 15–25 in defined conditions could differentiate into dopaminergic neurons in PA6-CM. As shown in Fig. 3, dopaminergic neuronal induction by PA6-CM was efficient, as numerous β-III tubulin^+^ neurons (A) and TH^+^ cells (B) were present in the cultures after 3 weeks of differentiation. The average percentage of TH^+^ neurons was 35±5% at day 20.

To further test if cells generated under these conditions were authentic midbrain dopaminergic neurons of the A9 type, we first examined the expression of additional midbrain dopaminergic markers in our cultures by immunocytochemistry. After 5 weeks of differentiation, a high percentage of the cells expressed Lmx1a, a transcription factor involved in dopaminergic development. Many TH^+^ neurons co-expressed Lmx1a (Fig. 3C). VMAT was also co-expressed in the majority of TH^+^ neurons (Fig. 3D). Importantly, Girk2, a marker of nigral neurons, was co-expressed in many TH^+^ neurons (Fig. 3E). The expression of a few other neuronal markers in our culture was also analyzed by immunostaining. We did not see DBH-positive cells in our culture and found a very small percentage of the cells (<1%) expressed GABA (Supplementary Figure S1). No glutamate or HB9 immunoreactive cells were found in the culture.

We then used real time PCR to examine the expression of a panel of 10 markers (En1, Otx2, Msx1, Nurr1, Lmx1b, AADC, Aldh1a, VMAT, DAT and Girk2) during dopaminergic differentiation (NSCs, day 16 and day 31). As seen in Fig. 3F, many of these genes (En1, Msx1, Nurr1, Lmx1b, AADC, VMAT, DAT and Girk2) were differentially upregulated in both day 16 and day 31 dopaminergic populations compared to the NSC population.

Finally we performed a large-scale microarray for gene expression profiling of the day 32 dopaminergic population. As summarized in Table 1, many midbrain and dopaminergic neuronal markers such as EN1, Pax2, Otx2, Msx1, Lmx1a, Ngn2 and Nurr1, transcription factors that were believed to be important in regulating dopaminergic differentiation, were detected in the day 32 dopaminergic population. These results indicate that our culture conditions can efficiently generate authentic A9 type neurons.

**Table 1:** Genes highly expressed in dopaminergic neurons by microarray analysis.

Markers	Gene name	NSCs	D32	D32/NSCs
NSC	*SOX1*	39	1	0.03
	*NES*	3509	2501	0.71
	*PAX6*	530	197	0.37
	*huD*	55	1465	26.64
Dopaminergic pathway	*EN1*	19	43	2.26
	*EN2*	281	16	0.06
	*OTX2*	1	82	82.00
	*PAX2*	3	97	32.33
	*MSX1*	40	152	3.80
	*LMX1A*	1	48	48.00
	*NEUROG2*	324	505	1.56
	*FOXA2*	1	94	94.00
	*GLI3*	1299	82	0.06
	*DVL3*	1334	450	0.34
	*SFRP2*	9352	285	0.03
	*TH*	11	700	63.64
	*DDC*	39	87	2.23
	*SMO*	890	108	0.12
	*KCNJ6*	6	797	132.83
	*Nurr1*	9	107	11.89
	*SRGAP3*	277	6425	23.19
	*RET*	9	36	4.00
	*FGF20*	9	44	4.89

**Figure 4:**
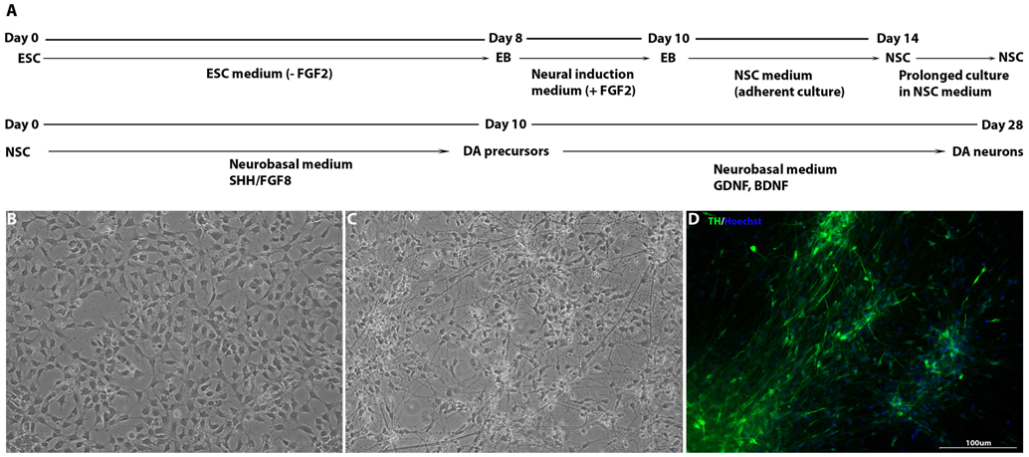
Four-step differention into midbrain dopaminergic neurons in a completely defined medium. (A) A schematic diagram of the four-step (ESC→propagation and storage of NSC→induction of a midbrain dopaminergic precursor population→maturation of the precursor to dopaminergic neurons) differentiation protocol for generation of dopaminergic neurons in completely defined media. (B–D) Defined medium adapted NSCs differentiated into dopaminergic neurons in defined media.

Since PA6 is a mouse stromal cell line and PA6-CM is not defined, in a separate set of experiments we identified a candidate set of factors that may substitute for PA6-CM in cultures (unpublished data). Given the present results, we tested whether the effects of PA6-CM could be mimicked with a combination of growth factors in a defined medium base. Our gene expression data and preliminary experiments indicated that Shh and FGF8 may substitute for PA6-CM as a source of dopaminergic inducing activity (Step 3: NSC→dopaminergic precursor) and that TGFβ, GDNF and BDNF may mimic the effect of dopaminergic neuron maturation and survival (Step 4: dopaminergic precursor→dopaminergic neuron). To test this, NSCs were exposed to Shh and FGF8 in a defined medium (NSC defined medium minus FGF2 with the addition of Shh and FGF8) for 10 days followed by 18 days of culture in the presence of TGFβ, GDNF and BDNF without Shh and FGF8, in the same basal serum-free defined medium (Fig. 4A). Under these conditions, approximately 25% of the cells expressed TH (Fig. 4B). The efficiency of this dopaminergic differentiation is comparable to that observed with exposure to PA6-CM for 3 weeks. These results indicate that Shh and FGF8 could replace PA6-CM in the step of dopaminergic induction from the NSC stage and that BDNF, GDNF and TGFβ were sufficient to maintain the differentiated population.

### Survival of dopaminergic neurons in vivo

**Figure 5:**
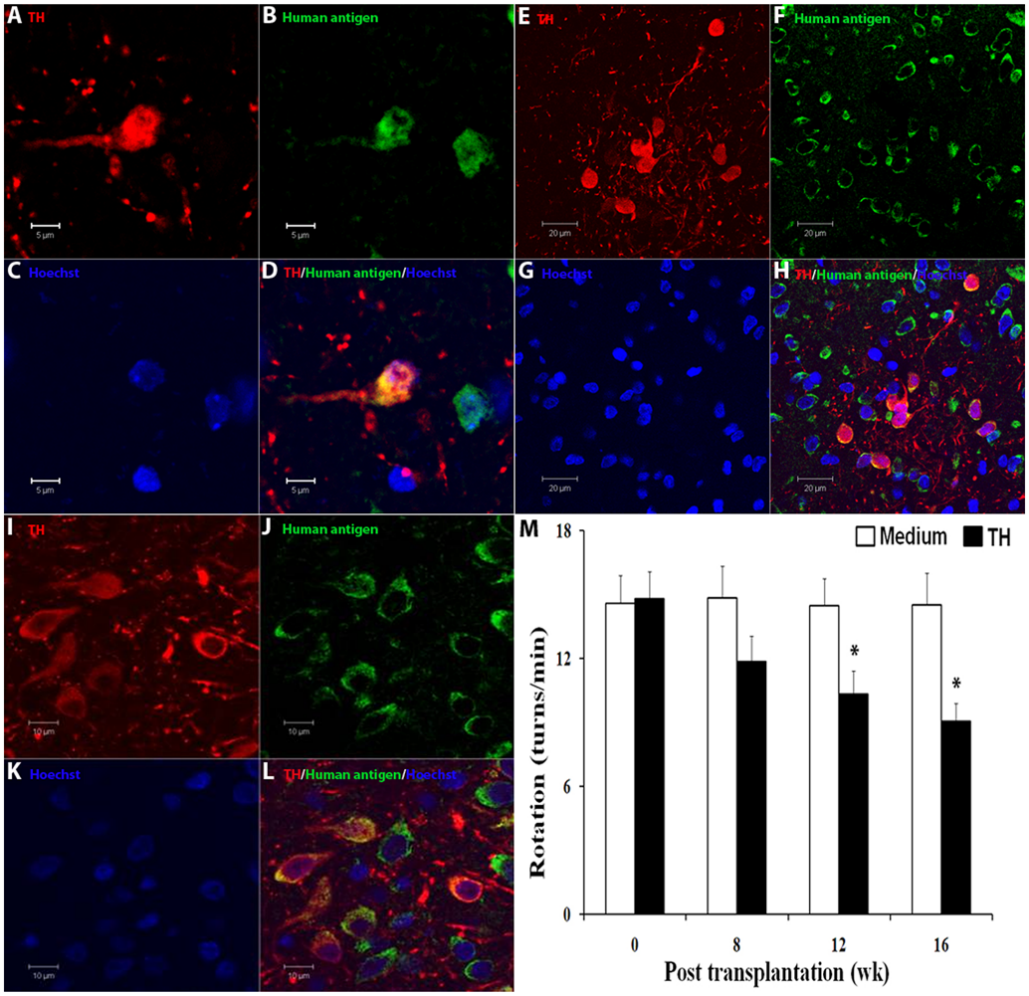
NSCs differentiated into functional dopaminergic neurons in vivo. Day 20 cells (after the NSC stage) were transplanted into the striatum of rats. Differentiation and survival of donor-derived dopaminergic neurons are seen in striatum two (A–D) and seven (E–H) weeks post of transplantation. Transplantation of day 20 cells in 6-OHDA rats showed a subset of human dopaminergic neurons, which survived eight weeks post of graft (I–L). Rotary tests showed amelioration of behavioral deficits in PD rats transplanted with donor-derived dopaminergic neurons (M).

To examine the functional properties of dopaminergic neurons derived from ESCs/NSCs cultured in defined medium, we transplanted cells that had been differentiated for 20 days (after the NSC stage) into the striatum of rats. Immunohistochemical analysis was performed 2 and 7 weeks after grafting and donor cells of human origin (positive for human nuclear antigen) were found in all brains throughout the graft sites (Fig. 5A–D). A small number of TH^+^ human cells were identified in the brain at the graft site 2 weeks post transplantation, and the numbers of TH^+^ human neurons were increased in brains 7 weeks after grafting (Fig. 5E–H). These results indicate that dopaminergic neurons generated from ESCs/NSCs cultured in defined medium could survive and integrate into the brain in vivo for at least 7 weeks, the duration of the present study. At 7 weeks post-transplantation, histological analyses showed that 2,579±581 cells/mm^3^ were TH-positive neurons among the 110,193±19,746 cells/mm^3^ in these grafts.

To further demonstrate that dopaminergic neurons grown in our culture conditions contained functional dopaminergic neurons, we transplanted the cells (20 days after the NSC stage) into eighteen 6-OHDA rats and showed that they can ameliorate behavioral deficits in PD rats. As seen in Fig. 5M, eight control rats that were transplanted with medium showed no attenuation of rotary behavior over the course of the experiment, whereas rats that were transplanted with grafts demonstrated significant rotational improvement at 12 and 16 weeks after transplantation (*p*<0.05). Human antigen-immunopositive cells co-expressing TH were found in all brains throughout the graft sites at 8 weeks following the transplantation (Fig. 5I–L).

### Completely serum- and xeno-free defined culturing of hESCs and NSCs

**Figure 6:**
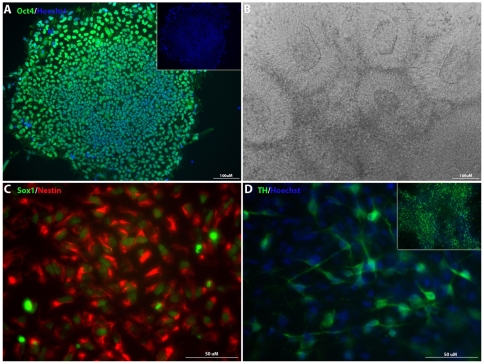
Completely serum- and xeno-free defined culturing of hESCs and NSCs. Morphology and expression of Oct4 (A) in hESCs that were cultured in xeno-free medium (hKSR with growth factors) for 7 passages. NSCs derived from xeno-free cultured hESCs expressed nestin and Sox1 (B–C) and could differentiate into dopaminergic neurons (D).

Examining our protocols we noted that while our simplified protocols for hESC culture, deriving NSCs, and inducing and maintaining dopaminergic neurons worked well, both the ESC medium and NSC medium used contained one xeno component, BSA. We therefore tested if cells could be grown in a completely humanized base medium with hKSR and the growth factors (Invitrogen) present in the defined medium, and could be differentiated appropriately. Fig. 6A shows an Oct4^+^ hESC colony cultured in this completely xeno-free defined medium for 7 passages. Similar to ESCs cultured in ESC defined medium with BSA, NSCs could be effectively generated from ESCs adapted to this completely xeno-free medium (Fig. 6B–C). Moreover, these NSCs retained the capacity to differentiate into dopaminergic neurons when exposure to PA6-CM or Shh and FGF8 (Fig. 6D). No apparent differences were found between NSCs generated under this xeno-free condition and the defined conditions described above in terms of dopaminergic differentiation efficiency. These results suggest that NSCs and dopaminergic neurons can be generated under xeno-free defined conditions from hESCs adapted to xeno-free defined medium and substrate, an important step for potential clinical applications of hESC-derived cells.

### Generation of dopaminergic neurons using hESC derived in defined, nearly xeno-free, conditions

**Figure 7:**
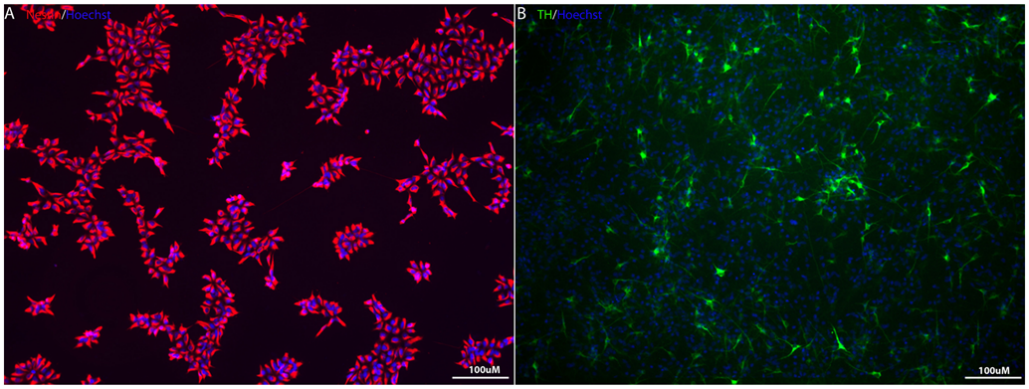
Generation of dopaminergic neurons from serum-free derived hESCs. NSCs could be generated from hESC line derived under serum-free and minimal xeno component exposure conditions as efficient as from defined medium adapted hESCs. Expression of nestin in NSCs (A) and differentiation into dopaminergic neurons (B).

We have previously shown that hESCs can be maintained in defined conditions for prolonged time periods while retaining their pluripotency, and our current experiments suggested that each of the remaining steps could utilize such a line to generate dopaminergic neurons. We reasoned that a new line isolated without exposure to xeno components could be used in such a differentiation process with our defined medium protocols, and that this line could generate clinically useful differentiated cell populations. We therefore tested a hESC line (W10) that was derived under serum- and nearly xeno-free conditions [30]. As seen in Fig. 7, nestin-expressing NSCs could be generated from W10 cells cultured in defined medium and these NSCs could be differentiated into TH^+^ dopaminergic neurons in a similar manner as the I6-derived NSCs.

## Discussion

In this manuscript we describe a process that is readily scalable for production of functional dopaminergic neurons from hESCs for potential therapeutic applications. This process can be transferred to a GMP facility that could generate a qualified product for clinical use. The process can be broken into four steps: 1) derivation and propagation of ESC, 2) propagation and storage of NSC, 3) induction of a midbrain dopaminergic precursor population, and 4) maturation of the precursor to dopaminergic neurons. Our data suggest that each step could utilize xeno-free defined media that are suitable for scalable GMP manufacture of cells for clinical use. Neurons generated by this process appear to be authentic A9 dopaminergic neurons as assessed by in vitro (expression of midbrain dopaminergic markers) and in vivo (ability to survive after transplantation) assays.

Although it is not necessary to exclude xeno components in the culture and differentiation of hESCs for clinical applications, provided appropriate validation tests are performed, we believe that xeno components should be avoided as much as possible, because exposure to animal products raises unique concerns for medical use [31]. Analyzing the components in our defined medium preparations, we identified three major sources of xeno material: BSA as a component of various media formulations, geltrex (which was used as a substrate in several stages of the process) and PA6-CM. In addition, there was the potential of exposure to xeno components as part of the hESC derivation process. We showed that hKSR (in completely humanized base medium with growth factors) could replace BSA and serum containing medium, and that a defined substrate - fibronectin (CellStart) - could substitute for geltrex in the culture of hESCs and hESC-derived NSC, and the process of inducing NSCs. Likewise, Shh and FGF8 could substitute for PA6-CM in the induction process and BDNF and GDNF along with TGFβ could substitute for the maintenance and survival effect of PA6-CM.

One advantage of our process of generating dopaminergic neurons from hESCs is the potential for scalability. As shown in the results, NSCs derived from defined cultured hESCs could be frozen and thawed, and NSCs cultured for prolonged periods retain the ability to differentiate into authentic A9 dopaminergic neurons both in vitro and in vivo. The efficiency of dopaminergic differentiation is sufficient for scalable production, as a high percentage of dopaminergic neurons can be obtained without enrichment.

A process amendable for GMP manufacture does not simply require the development of components, but also replicable tests to establish the quality of the end product. Toward this end, we have employed a large-scale microarray analysis and identified a panel of stage-specific markers that could be used to rapidly assess the quality of the end product. These include markers of NSCs, dopaminergic precursors and mature dopaminergic neurons, expression of which could be reliably assessed by real time PCR and/or immunocytochemistry.

Although the protocol we described works with multiple hESC lines, and we and others have shown that multiple lines can be adapted to serum free conditions, we acknowledge that we have not yet successfully derived a line in completely xeno-free defined conditions and shown that we can successfully generate dopaminergic neurons using this process. As an interim measure we have adapted a hESC line derived under nearly xeno-free condition [30] to defined medium conditions using CellStart as a substrate at an early passage culture. We show that such an adapted line can be readily differentiated into dopaminergic neurons using our four-step protocol and the defined media and growth factors described. We believe that these results suggest that it will be possible to derive cell lines in hKSR on a defined substrate and that such lines will be similar to other hESC lines and will differentiate into dopaminergic neurons following this protocol.

We note as well that we have used growth factors and components that were themselves not manufactured under GMP protocols as would be required for a true GMP process, nor was this process tested in a GMP facility. In addition, we have not demonstrated a GMP-applicable process for purifying dopaminergic neuron cultures. There were several reasons why we did not undertake these efforts even though we believe that these are easily solvable problems. One important reason was cost, as developing GMP grade material such as antibodies and growth factors would be required prior to finalizing a protocol. The second was the lack of access to a GMP facility that had the requisite expertise in adherent cell culture and dopaminergic neuron differentiation, and the absence of consensus on what degree of purification (if any) is required for a final product. We have however initiated preliminary discussions with commercial providers who have assured us that such products can be developed when they are required.

In summary, we have shown that hESCs and NSCs can be maintained in xeno-free defined media for a prolonged period of time while retaining their ability to differentiate into authentic dopaminergic neurons. Our defined medium system provides a path to a scalable GMP-applicable process of generation of dopaminergic neurons from hESCs for therapeutic applications, and a ready source of large numbers of neurons for potential screening applications.

## Supporting Information

Figure S1 Only a small fraction (<1%) of neuronal population differentiated for 31 days in PA6 CM shows positive GABA staining.
(3.63 MB TIF)

Table S1 Sequences of primers used for qRT-PCR analysis
(0.01 MB DOC)
