# Supplementary figures and images for: Xeno-Free Defined Conditions for Culture of Human Embryonic Stem Cells, Neural Stem Cells and Dopaminergic Neurons Derived from Them
Source: PLoS One. 2009 Jul 14;4(7):e6233. doi: 10.1371/journal.pone.0006233 (PMC2705186; doi:10.1371/journal.pone.0006233)

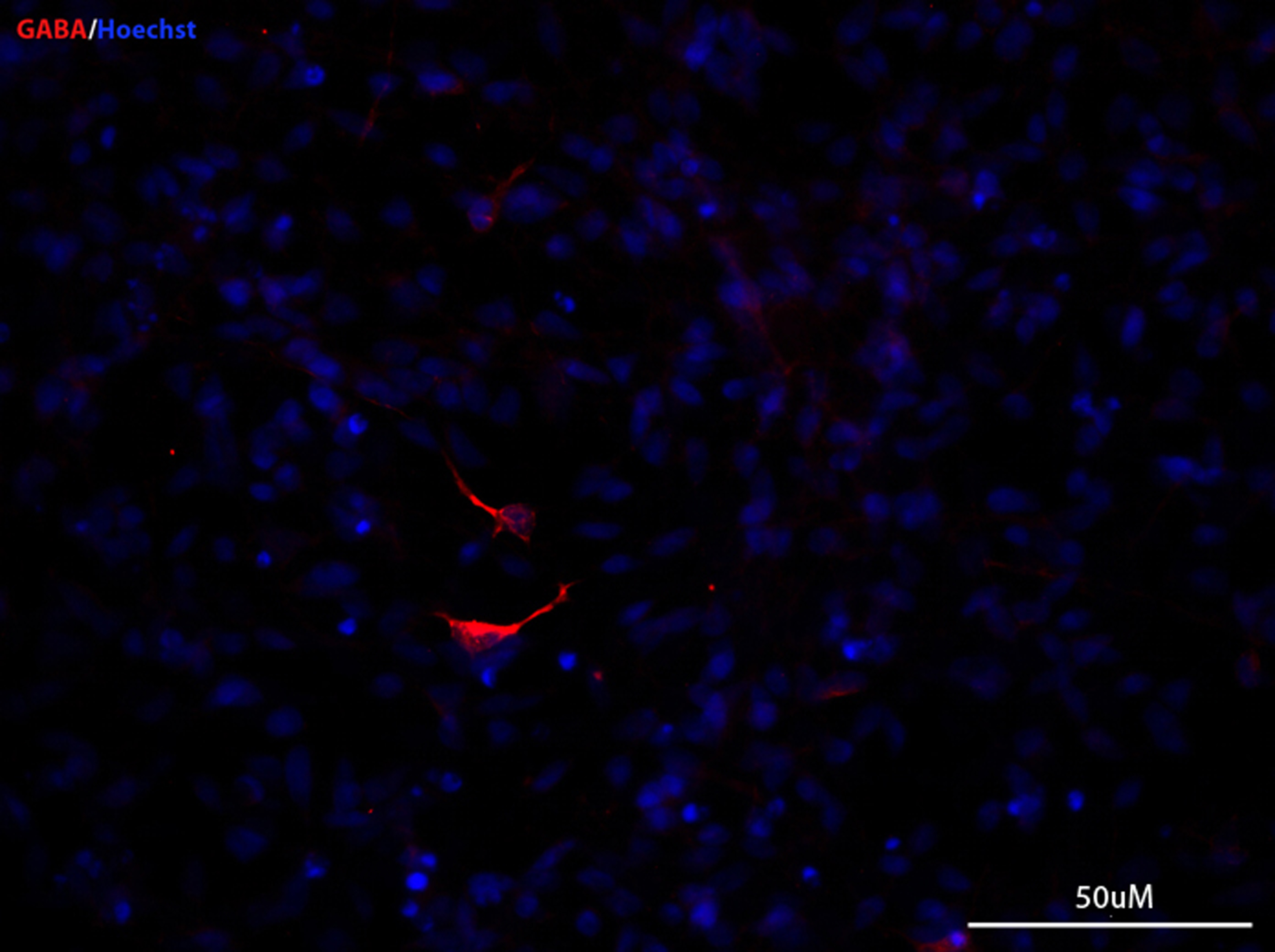

Supplement: Figure S1 — Only a small fraction (<1%) of neuronal population differentiated for 31 days in PA6 CM shows positive GABA staining. (3.63 MB TIF) [file pone.0006233.s001.tif]
