# Supplementary material for: Xeno-Free Defined Conditions for Culture of Human Embryonic Stem Cells, Neural Stem Cells and Dopaminergic Neurons Derived from Them
Source: PLoS One. 2009 Jul 14;4(7):e6233. doi: 10.1371/journal.pone.0006233 (PMC2705186; doi:10.1371/journal.pone.0006233)
Supplement: Table S1 — Sequences of primers used for qRT-PCR analysis (0.01 MB DOC) [file pone.0006233.s002.doc]

| Nurr1-F | ATTAGCATACAGGTCCAACCC |
| --- | --- |
| Nurr1-R | TGGAGGAGAATTCAACAATGG |
| VMAT-F | TGGATTCGTCAATGATGCCT |
| VMAT-R | CAGAAGGACCTATAGCATACCC |
| DAT-F | CCTGCTCTTCATGGTCATTG |
| DAT-R | AAGCCCACACCTTTCAGTAT |
| TH-F | GTGCTAAACCTGCTCTTCTC |
| TH-R | GCTTCAAACGTCTCAAACAC |
| AADC-F | CAATCTCTTAGAAGTCGGTCCT |
| AADC-R | AATCTGCAAACTCCACTCCA |
| Msx1-F | AGACGCAGGTGAAGATATGG |
| Msx1-R | ATCTTCAGCTTCTCCAGCTC |
| Gapdh-F | CAAGATCATCAGCAATGCCT |
| Gapdh-R | CTTCCACGATACCAAAGTTGTC |
| En1-F | GCCAAGATCAAGAAAGCCAC |
| En1-R | TACTCGCTCTCGTCTTTGTC |
| Girk2-F | CATGATTGAGTGAAGCCACC |
| Girk2-R | AGGACGTTAGTCATGGATTCTG |
| Otx2-F | GTACCCAGACATCTTCATGC |
| Otx2-R | GATTCTTAAACCATACCTGCACC |
| Lmx1b-F | ACAGCGATACCTCCTTAACC |
| Lmx1b-R | AACTCTGCATGGAGTAGAGC |

Sup Table 1. Sequences of primers used for qRT-PCR analysis
